# Flavonoids from the Roots of *Sophora flavescens* and Their Potential Anti-Inflammatory and Antiproliferative Activities

**DOI:** 10.3390/molecules28052048

**Published:** 2023-02-22

**Authors:** Yan-Fei Yang, Ting-Ting Liu, Guo-Xian Li, Xuan-Qin Chen, Rong-Tao Li, Zhi-Jun Zhang

**Affiliations:** Faculty of Life Science and Technology, Kunming University of Science and Technology, Kunming 650500, China

**Keywords:** *Sophora flavescens*, flavonoids, cyclohexanoid prenylflavonoids, NO production inhibitory activity, antiproliferative

## Abstract

The phytochemical investigation of the roots of the traditional Chinese medicinal plant *Sophora flavescens* led to the isolation of two novel prenylflavonoids with an unusual cyclohexyl substituent instead of the common aromatic ring B, named 4′,4′-dimethoxy-sophvein (**17**) and sophvein-4′-one (**18**), and 34 known compounds (**1–16**, **19–36**). The structures of these chemical compounds were determined by spectroscopic techniques, including 1D-, 2D-NMR, and HRESIMS data. Furthermore, evaluations of nitric oxide (NO) production inhibitory activity against lipopolysaccharide (LPS)-treated RAW264.7 cells indicated that some compounds exhibited obvious inhibition effects, with IC_50_ ranged from 4.6 ± 1.1 to 14.4 ± 0.4 μM. Moreover, additional research demonstrated that some compounds inhibited the growth of HepG2 cells, with an IC_50_ ranging from 0.46 ± 0.1 to 48.6 ± 0.8 μM. These results suggest that flavonoid derivatives from the roots of *S. flavescens* can be used as a latent source of antiproliferative or anti-inflammatory agents.

## 1. Introduction

The roots of *Sophora flavescens* are ordinarily served in the traditional Chinese medicine (TCM), “Ku Shen”, for the curing of skin diseases, cancer, dysentery, hematochezia, jaundice, pruritus vulvae, eczema, and hepatitis [1]. Modern pharmacological research shows that it exhibits outstanding activities toward tumors, inflammation, diabetes, and microbial infections [2,3,4,5,6,7]. Phytochemical studies demonstrated that alkaloids and flavonoids are the major chemical classes of *S. flavescens* compounds [8,9,10]. Matrine, as a reflective component of the alkaloids of *S. flavescens*, has been used as an antitumor drug (Compound matrine injection) in China. With more in-depth research on the alkaloids of *S. flavescens*, the anticancer effects of some alkaloids have been shown to be more potent than those of flavonoids of *S. flavescens* in vitro and in vivo [11].

**Figure 1:**
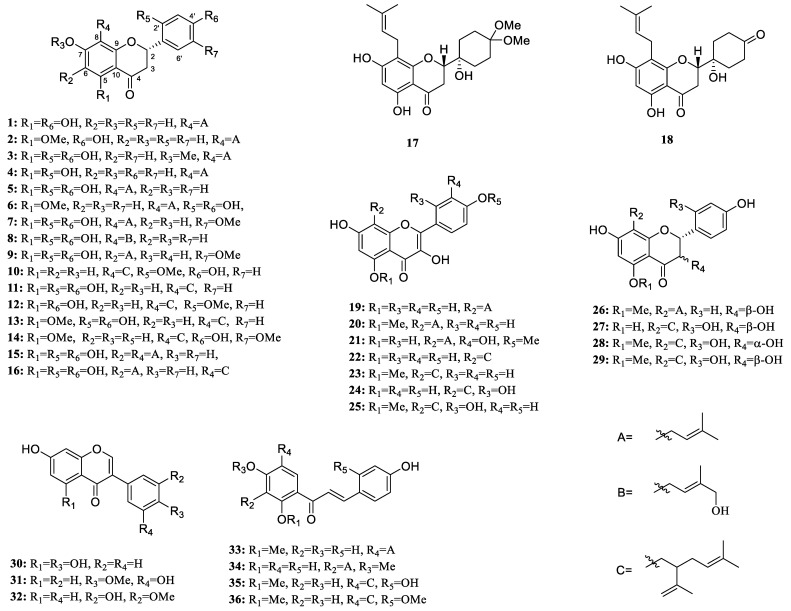
Chemical structures of isolated compounds.

In order to discover new compounds with antiproliferative and anti-inflammatory activities from *S. flavescens*, we isolated 36 flavonoids from this medicinal plant (Figure 1), including flavanones, isoflavones, flavonols, flavanonols, and chalcones. Among them, compounds **17** and **18** were two new dihydroflavones with an unusual cyclohexyl substituent instead of the common aromatic ring B. Many studies have shown an interesting link between chronic inflammation and cancers [12]. Thus, all isolated components were evaluated for their anti-inflammatory activity by acting on LPS-stimulated macrophage (RAW 264.7) cell lines in vitro. In addition, all components were assessed for their antiproliferative activities against HepG2 cell lines.

Here, we characterize the isolation, chemical structure elucidation, antiproliferative activity against HepG2 cells, and NO production inhibitory activity of all isolates.

## 2. Results and Discussion

The comprehensive use of separation materials and chromatographic methods such as normal silica gel, hydroxypropyl dextran gel (Sephadex LH-20), MPLC, TLC, MCI reversed-phase column, and HPLC, with the help of MS and NMR and other spectroscopy methods, was carried out to isolate and identify 36 compounds, including 2 new ones: 4′,4′-dimethoxy-sophvein (**17**) and sophvein-4′-one (**18**). 

### 2.1. Structural Elucidation

Compound **17** was acquired as a white amorphous powder. The HR-ESI-MS demonstrated the molecular ion peak at *m/z* 429.1894 [M+Na]^+^ (calcd. for 429.1884), corresponding to the molecular formula C_22_H_30_O_7_, which indicated eight degrees of unsaturation. The ^1^H spectroscopic data of **17** (Table 1) demonstrated the existence of one aromatic proton signal (*δ*_H_ 5.84 (1H, s)), one isopentenyl side chain signal (*δ*_H_ 1.68 (3H, s); 1.59 (3H, s); 3.14 (2H, d, *J* = 6.9 Hz); 5.11 (1H, br t, *J* = 6.9 Hz)), and two methoxy groups signals (*δ*_H_ 3.15 (3H, s) and 3.10 (3H, s)). By interpreting the DEPT and ^13^C NMR spectra, 22 carbon signals (Table 1) were observed, including two methoxy groups (*δ*_C_ 47.9 and 48.1), two methyls (*δ*_C_18.1 (C-5″) and 25.9 (C-4″)), three methylidynes (*δ*_C_ 84.6 (C-2); 96.4 (C-6); 124.4 (C-2″)), six methylenes (*δ*_C_ 22.4 (C-1″); 28.4 (C-3′); 28.5 (C-5′); 29.9 (C-2′); 31.3 (C-6′); 37.0 (C-3)), three oxygenated aromatic carbons (*δ*_C_161.3 (C-9); 163.0 (C-5); 166.0 (C-7)), and six quaternary carbons (*δ*_C_ 72.2(C-1′); 101.1(C-4′); 103.2 (C-10); 109.0 (C-8); 131.6 (C-3″); 198.8 (C-4)). According to the above NMR data, it is speculated that this compound may be an isopentenyl substituted flavanone. However, from the ^1^H NMR spectra of compound **17**, the proton signal of the typical aromatic flavonoid B-ring in the flavonoid compound completely disappeared, and a complex signal of eight aliphatic protons emerged instead.

**Figure 2:**
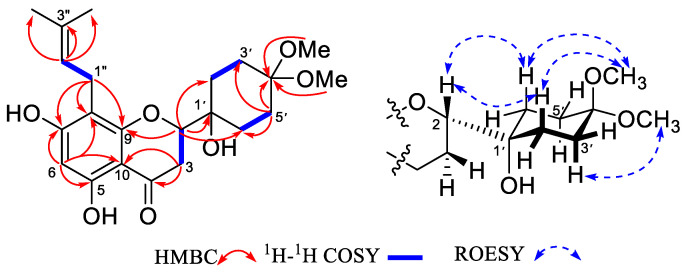
The structure and the key 2D NMR correlations of **17**.

These data suggest the presence of a hydrogenated B-ring unit in the compound, and this inference is also confirmed by the HMQC and HMBC spectra (Figure 2). Comparing the ^13^C NMR data, except for the isopentenyl signal, the remaining 17 signals are very similar to (2*S*)-4′,4′-dimethoxy-ongokein [13]. The main difference between the two compounds is that the H-8 (*δ*_H_ 5.98 (d, *J* = 2.0 Hz)) signal in (2*S*)-4′,4′-dimethoxy-ongokein disappears, and the chemical shift of C-8 also decreases to the lower field. These data indicate that the isopentenyl group is attached at the C-8 position. This inference was identified via the HMBC correlation between H-1″ (*δ*_H_ 3.14, 2H, d, *J* = 6.9 Hz) and C-9 (*δ*_C_ 161.3), C-8 (*δ*_C_ 109.0), and C-7 (*δ*_C_ 166.0). On the other hand, the attachment of the two methoxy groups to the C-4′-position was shown by the HMBC correlation peaks between two methoxy signals (*δ*_H_ 3.15 and 3.10) and C-4′ (*δ*_C_ 101.1).

By comparison, the coupling constants of H-2 (*δ*_H_ 4.04 (d, *J* = 13.6 Hz)), H-3a (*δ*_H_ 2.81 (dd, *J* = 17.0, 13.6 Hz)), and H-3b (*δ*_H_ 2.51 (d, *J* = 17.0 Hz)) were found to be very close to (2*S*)-4′,4′-dimethoxy-ongokein [13], so it is inferred that the C-2 of compound **17** is in the *S* configuration. Additionally, the configuration of the B-ring of cyclohexane was defined to be the _1′_C^4′^ configuration via the NOE effect in the ROESY experiment (Figure 2). According to the above analysis, the structure of compound **17** was confirmed and named 4′,4′-dimethoxy-sophvein.

Compound **18** was acquired as a white amorphous powder. The HR-ESI-MS displayed the molecular ion peak at *m/z* 361.1650 [M+H]^+^ (calcd for 361.1646), suggesting the molecular formula C_20_H_24_O_6_ with 9° of unsaturation. The ^1^H and ^13^C NMR spectroscopic data of **18** are similar to **17** (Table 1), with the main difference being the disappearance of the two methoxyl signals in **18**. Furthermore, the chemical shift of C-4′ exhibited a downfield shift (from *δ*_C_ 101.1 to 210.3). Combined with HR-ESI-MS, it was found that the molecular weight of compound **18** is 46 less than that of **17**. The above data indicate that the C-4′ of the B-ring of compound **18** has a carbonyl group. This inference was further defined by the HMBC correlation of the *δ*_H_ 2.03 (H-3′b), 2.21 (H-2′), and 2.10 (H-6′) proton signals with C-4′ (*δ*_C_ 210.3). Finally, by comparing the NOE effects in the ROESY spectra of the two compounds, it was found that **18** and **17** have the same configuration. The optical rotation values of the two compounds [${\left[\alpha \right]}_{D}^{24}$ = + 49.88 (*c* 0.25, MeOH) for **17** and ${\left[\alpha \right]}_{D}^{24}$ = +38.69 (*c* 0.29, MeOH)] for **18** also support this inference. Based on the above data analysis, the structure of **18** was confirmed and named sophvein-4′-one. See Supplementary Materials for HR-ESI-MS, ^1^H and ^13^C NMR, HMQC, HMBC, ^1^H-^1^H COSY, and ROESY of compounds **17** and **18**.

The known compounds (**1**–**16** and **19**–**36**) were determined based on a comparison with published NMR data in the references to be sophorafiavanone B (**1**) [14], isoxanthohumol (**2**) [15], kenusanonoe I (**3**) [16], kushenol S (**4**) [17], leachianone G (**5**) [18], 5-methoxy-7,2′,4′-trihydroxy-8-[3,3-dimethyl-allyl]flavanone (**6**) [19], kushenol W (**7**) [17], 8-(3-Hydroxymethyl-2-butenyl)-5,7,2′,4′-tetra-hydroxyflavanone (**8**) [20], kushenol V (**9**) [17], alopecurone G (**10**) [21], sophoraflavone G (**11**) [22], leachianone A (**12**) [23], kurarinone (**13**) [24], (2*S*)-2′-methoxykurarinone (**14**) [25], kushenol E (**15**) [26], kushenol B (**16**) [26], noranhyoicaritin (**19**) [27], sophoflavescenol (**20**) [28], 8-(3,3-dimethylallyl)-tamarixetin (**21**) [29], 8-lavandulylkaempferol (**22**) [30], kushenol Z (**23**) [31], kushenol C (**24**) [32], 5-*O*-methylkushenol C (**25**) [33], (2*R*)-3*β*,7,4′-trihydrox-y-5-methoxy-8-prenyl- flavanone (**26**) [20], kushenol X (**27**) [23], kushenol N (**28**) [34], kushenol I (**29**) [35], 5,7,4′-trihydroxyisoflavone (**30**) [36], 7,3′-dihydroxy-4′-methoxyisoflavanone (**31**) [37], calycosin (**32**) [36], xanthohumol (**33**) [38], xanthogalenol (**34**) [39], kuraridin (**35**) [34], and kushenol D (**36**) [40].

### 2.2. Biological Studies

The potential cytotoxicity of compounds on RAW 264.7 macrophages cells was determined before executing further studies. Macrophage cells were treated with compounds, and a mitochondria colorimetric (MTT) assay was used to test cell survival. Cell viability (%) = [(O.D_Drug_ − O.D_Blank_)/(O.D_Control_ − O.D_Blank_)] ×100%, and SPSS version 21.0 (probit analysis) was used for calculating CC_50_ extract. Table 2 shows that some compounds exhibited obvious cytotoxicity on macrophages. Among them, compound **22** was the most toxic composition with a CC_50_ value of 13.8 ± 0.6 μM.

NO is one of the immune effectors used by macrophages to defend our bodies against intracellular pathogens. To determine if compounds **1**–**36** can modulate the NO production by macrophages, the Griess reagent was used to analyze the NO levels. The cells were incubated with compounds **1**–**36** (3.12, 6.25, 12.5, 25, and 50 μM) and then treated with LPS for 24 h. When activated by LPS, NO was produced from macrophages after inducing iNOS genes and subsequent protein expression. NO production inhibition (%) = [(O.D_LPS_ − O.D_Drug_)/(O.D_LPS_ − O.D_Blank_)] × 100%. The determination of IC_50_ was calculated using probit analysis in SPSS version 21.0. The research results show that some compounds exhibited obvious NO production inhibitory activity, with IC_50_ ranging from 4.6 ± 1.1 to 14.4 ± 0.4 μM, as shown in Table 2. Among them, compound **35** displayed the most significant inhibition of NO production, with an IC_50_ value of 4.6 ± 1.1 μM.

The inhibitory rate of HepG2 cells growth of compounds **1**–**36** was measured by the MTT assay and compared with a negative control and positive control (cisplatin, IC_50_ = 24.5 ± 0.8 μM). Inhibitory rate (%) = [(O.D_Drug_ − O.D_Blank_)/(O.D_Control_ − O.D_Blank_)] ×100%. The IC_50_ values were calculated with probit analysis using software SPSS version 21.0. The results show that eight compounds inhibited the growth of HepG2 cells with an IC_50_ ranging from 0.46 ± 0.1 to 48.6 ± 0.8 μM (Table 3), while others were inactive (IC_50_ > 50 μM). Compound **22** exhibited the best antiproliferative effects on HepG2 cell lines, with an IC_50_ value of 0.46 ± 0.1 μM. The present work revealed that many secondary metabolites of *S*. *flavescens* have antiproliferative and NO production inhibitory activities, indicating its application in traditional Chinese medicine.

## 3. Materials and Methods

### 3.1. General Experimental Procedure

The optical rotation value was recorded with a Jasco DIP-370 polarimeter (JASCO Corporation, Tokyo, Japan). The ultraviolet (UV) spectrum was recorded by a UV2700 spectrophotometer (Shimadzu, Kyoto, Japan). The infrared (IR) spectrum was obtained by an FT-IR spectrophotometer (PerkinElmer, Waltham, MA, USA) using KBr pellets. High-resolution electrospray ionization mass spectroscopy (HRESIMS) was obtained with an Agilent 6500 LC/Q-TOF mass spectrometer (Agilent, Waldbronn, Germany). The ^1^H NMR, ^13^C NMR, HMBC, HSQC, ^1^H-^1^H COSY, and ROESY spectra were measured by a Bruker Advance III HD using tetramethylsilane (TMS) as an internal standard (600 MHz, Bruker BioSpin, Zürich, Switzerland). Chemical shifts are demonstrated in *δ* (ppm) and relative to the residual solvent signals. Silica gel (100–200 and 200–300 mesh, Qingdao Marine Chemical Industry Co., Qingdao, China), polyamide (60–90 mesh, Changfeng Chemical Factory Co., Gulou, Nanjing, China), sephadex LH-20 (Sigma-Aldrich Corp., St. Louis, MO, USA), and RP-18 reverse-phase silica gel (20–45 µm, Fuji Silysia Chemical Ltd., Kasugai-shi, Japan) have been used for column chromatography (CC). The reverse-phase medium-pressure liquid chromatography (RP-MPLC) system comprises a Büchi pump (Büchi Labortechnik AG, Meierseggstrasse 40 Postfach CH-9230, Flawil, Switzerland), column and precolumn (310 × 36 mm and 110 × 36 mm, Soochow high tech chromatography CO., Ltd., Suzhou, China), and MCI-gel CHP-20P (75–150 µm, Mitsubishi Chemical Co., Tokyo, Japan). Thin-layer chromatography (TLC) was performed using precoated silica gel plates (GF254, Qingdao Marine Chemical Factory, Qingdao, China), and the spots were visualized by spraying with 10% sulfuric acid ethanolic solution or α-naphthol-sulfuric acid solution and heating at 110 °C for 3–5 min. Murine macrophage (RAW 264.7) cells were acquired from Kunming Institute of Zoology (KIZ), Chinese Academy of Sciences (CAS). Macrophage cells were placed in a constant temperature incubator and cultured at a temperature of 37 °C and a concentration of 5% CO_2_. Human hepatoma HepG2 cell lines were also obtained from the Kunming Institute of Zoology (KIZ), Chinese Academy of Sciences (CAS). HepG2 cells were cultured at 37 °C under 5% CO_2_. Additionally, an inverted phase contrast microscope was used to observe cell morphology.

### 3.2. Plant Material

The roots of *S*. *flavescens* were collected in Honghe, Yunnan Province, People’s Republic of China, in June 2019. The sample was identified by one of the authors (Xuan-Qin Chen). A voucher specimen (number: KUMST20190628) was conserved at the Key Laboratory of Phytochemistry, Kunming University of Science and Technology, China.

### 3.3. Extraction and Isolation

The air-dried roots of *S*. *flavescens* (20 Kg) were crushed and extracted with 95% aq. ethanol (24 h × 3 times). The ethanol extracts were percolated and evaporated in vacuo to obtain a residue. This residue was suspended in water and then extracted with ethyl acetate (3 times) and concentrated to produce an ethyl acetate phase (493 g). The ethyl acetate phase was segmented and enriched by macroporous resin to obtain 308 g of total flavonoids. The total flavonoid extract (300 g) was segmented using a normal phase silica gel column chromatography (CC) and stepwise gradient elution with a gradient of petroleum ether-ethyl acetate (4:1–1:1) to obtain 7 subfractions (Fr.1–7).

Fr.1 (3.4 g) was further segmented by a silica gel chromatography column (CC) stepwise gradient elution with petroleum ether-dichloromethane (1:2) to yield two subfractions (F1-1~F1-2). F1-1 (1.9 g) was further segmented via Sephadex LH-20 (methanol) to obtain 8 subfractions (F1-1-1~F1-1-8). F1-1-5 (120.6 mg) was segmented by CC (silica gel) stepwise gradient elution with dichloromethane-methanol (150:1) to obtain compound **3** (14.0 mg). F1-2 (567.0 mg) was further segmented via Sephadex LH-20 (methanol) to obtain 8 subfractions (F1-2-1~F1-2-8). F1-2-5 (59.0 mg) was segmented by CC (silica gel) stepwise gradient elution with petroleum ether-ethyl acetate (9:1–2:1) to obtain compounds **1** (141.4 mg) and **4** (33.2 mg). F1-2-8 (80.0 mg) was segmented by CC (silica gel) and stepwise gradient elution with petroleum ether-ethyl acetate (10:1–5:1) to yield compound **22** (30.0 mg).

Fr.2 (1.5 g) was further segmented via Sephadex LH-20 (methanol) to obtain 6 subfractions (F2-1~F2-6). F2-4 (366.0 mg) was segmented by CC (silica gel) stepwise gradient elution with dichloromethane-methanol (150:1) to obtain 5 subfractions (F2-4-1~F2-4-5). F2-4-4 (28.0 mg) was further segmented via Sephadex LH-20 (methanol) to obtain compounds **15** (8.3 mg) and **16** (7.5 mg). F2-4-5 (15.0 mg) was further segmented via Sephadex LH-20 (methanol) to obtain compound **11** (6.0 mg). F2-6 (246.0 mg) was further segmented by CC (silica gel) stepwise gradient elution with dichloromethane-methanol (100:1–50:1) to obtain 5 subfractions (F2-6-1~F2-6-5). F2-6-3 (32.5 mg) was further segmented via Sephadex LH-20 (methanol) to yield compound **19** (24.3 mg). F2-6-5 (508.6 mg) was further segmented via Sephadex LH-20 (methanol) to obtain compound **21** (468.8 mg).

Fr.3 (30.0 g) was further segmented by CC (silica gel) stepwise gradient elution with dichloromethane-methanol (50:1–10:1) to obtain 2 subfractions (F3-1~F3-2). F3-1 (4.0 g) was further segmented via Sephadex LH-20 (methanol) to yield 7 subfractions (F3-1-1~F3-1-7). F3-1-3 (501.6 mg) was segmented by CC (silica gel) stepwise gradient elution with dichloromethane-methanol (50:1–20:1) to obtain 4 subfractions (F3-1-3-1~F3-1-3-4). F3-1-3-2 (61.2 mg) was segmented by CC (silica gel) stepwise gradient elution with dichloromethane-ethyl acetate (30:1–10:1) to obtain compound **10** (5.6 mg). F3-1-4 (1.1 g) was segmented by CC (silica gel) stepwise gradient elution with dichloromethane-methanol (50:1–10:1) to obtain 6 subfractions (F3-1-4-1~F3-1-4-6). F3-1-4-5 (642.5 mg) was segmented by CC (silica gel) stepwise gradient elution with dichloromethane-ethyl acetate (30:1–10:1) to obtain 3 subfractions (F3-1-4-5-1~F3-1-4-5-3). F3-1-5 (2.3 g) was segmented by CC (silica gel) stepwise gradient elution with dichloromethane-ethyl acetate (50:1–30:1) to obtain 6 subfractions (F3-1-5-1~F3-1-5-6). F3-1-5-1 (400.4 mg) was further segmented via Sephadex LH-20 (methanol) to obtain 2 subfractions (F3-1-5-1-1~F3-1-5-1-2). F3-1-5-1-1 (119.2 mg) was segmented by CC (silica gel) stepwise gradient elution with petroleum ether-acetone (4:1–1:1) to obtain compound **34** (27.8 mg). F3-1-5-1-2 (70.3 mg), followed by semipreparative high-performance liquid chromatography (HPLC) (70%, MeOH-H_2_O, 3 mL/min), was used to obtain compound **31** (4.1 mg). F3-2 (25.0 g) was first subjected to reverse-phase RP-18 chromatography with 40–100% methanol-water as the eluent, and the same fractions were combined by thin-layer chromatography to obtain 8 subfractions (F3-2-1~F3-2-8). F3-2-5 (276.5 mg) was further segmented via Sephadex LH-20 (methanol) to yield compound **30** (140.2 mg).

Fr.4 (11.0 g) was segmented by CC (silica gel) stepwise gradient elution with dichloromethane-methanol (60:1–10:1) to obtain 6 subfractions(F4-1~F4-6). F4-3 (900.7 mg) was further segmented via Sephadex LH-20 (methanol) to obtain 5 subfractions (F4-3-1-F4-3-5). F4-3-4 (163.3 mg) was segmented by CC (silica gel) stepwise gradient elution with dichloromethane-ethyl acetate (20:1–5:1) to obtain compound **36** (68.2 mg). F4-3-5 (78.5 mg) was segmented by CC (silica gel) stepwise gradient elution with dichloromethane-ethyl acetate (20:1–5:1) to obtain compound **33** (30.1 mg). F4-4 (900.6 mg) was further segmented via Sephadex LH-20 (methanol) to obtain 3 subfractions (F4-4-1~F4-4-3). F4-4-1 (20.3 mg) was segmented by CC (silica gel) stepwise gradient elution with petroleum ether-ethyl acetate (3:1) to obtain compound **17** (9.0 mg). F4-4-2 (102.3 mg) was segmented by CC (silica gel) stepwise gradient elution with petroleum ether-ethyl acetate (3:1) to obtain compound **18** (12.2 mg). F4-4-3 (500.1 mg) was segmented by CC (silica gel) stepwise gradient elution with chloroform-methanol (80:1–20:1) to obtain 4 subfractions (F4-4-3-1~F4-4-3-4). F4-5 (1.1 g) was further segmented via Sephadex LH-20 (methanol) to obtain 5 subfractions (F4-5-1~F4-5-5). F4-5-5 (350.4 mg) was segmented by CC (silica gel) stepwise gradient elution with petroleum ether-ethyl acetate (4:1–1:1) to obtain 4 subfractions (F4-5-5-1~F4-5-5-4). F4-5-5-1 (60.3 mg) was segmented by CC (silica gel) stepwise gradient elution with trichloromethane-methanol (60:1–10:1) to obtain compound **7** (27.5 mg). F4-5-5-3 (200.3 mg) was further segmented via Sephadex LH-20 (methanol) to obtain compound **9** (123.9 mg). F4-6-4 (2.1 g) was segmented by CC (silica gel) stepwise gradient elution with petroleum ether-ethyl acetate (4:1–1:1) to obtain 2 subfractions (F4-6-4-1~F4-6-4-2). F4-6-4-1 (1.2 g) was further segmented via Sephadex LH-20 (methanol) to obtain compound **27** (20.7 mg). F4-6-4-2-2 (1.0 g) was segmented by CC (silica gel) stepwise gradient elution with petroleum ether-ethyl acetate (3:1–1:1), and then was further segmented via Sephadex LH-20 (methanol) to obtain compounds **5** (60.6 mg) and **35** (40.2 mg).

Fr.5 (92.0 g) was passed through a normal-phase silica gel column, firstly subjected to reversed-phase RP-18 silica gel eluting with methanol-water (60–100%) to obtain 11 subfractions (F5-1~F5-11). F5-1 (1.1 g) was further segmented via Sephadex LH-20 (methanol) to obtain 4 subfractions (F5-1-1~F5-1-4). F5-2 (2.0 g) was further segmented via Sephadex LH-20 (methanol) to obtain 2 subfractions (F5-2-1~F5-2-2). F5-2-1 (90.8 mg) was repeatedly recrystallized to produce compound **32** (10.3 mg). F5-2-2 (1.3 g) was segmented by CC (silica gel) stepwise gradient elution with petroleum ether-acetone (2:1–1:1) to obtain 5 subfractions (F5-2-2-1~F5-2-2-5). F5-2-2-3 (201.4 mg) was segmented by CC (silica gel) stepwise gradient elution with dichloromethane-methanol (25:1–10:1) and then was further segmented via Sephadex LH-20 (methanol) to obtain compound **8** (5.0 mg). F5-3 (10.0 g) was segmented by CC (silica gel) stepwise gradient elution with trichloromethane-methanol (80:1–30:1) to obtain 6 subfractions (F5-3-1~F5-3-6). F5-3-3-7 (91.5 mg), followed by semipreparative HPLC (55%, MeOH-H_2_O, 3 mL/min), was used to obtain compound **2** (4.0 mg). F5-3-6 (5.0 g) was segmented by CC (silica gel) stepwise gradient elution with petroleum ether-acetone (3:1–1:1) to obtain 4 subfractions (F5-3-6-1~F5-3-6-4). F5-3-6-3, (76.1 mg) followed by semipreparative HPLC (50%, MeOH-H_2_O, 3 mL/min), was used to obtain compound **26** (4.2 mg). F5-3-6-4 (47.6 mg), followed by semipreparative HPLC (55%, MeOH-H_2_O, 3 mL/min), was used to obtain compound **28** (4.2 mg) and **29** (4.0 mg). F5-4 (2.0 g) was further segmented via Sephadex LH-20 (methanol) to obtain 9 subfractions (F5-4-1~F5-4-9). F5-4-1 (614.5 mg) was segmented by CC (silica gel) stepwise gradient elution with petroleum ether-acetone (3:1–1:1) to obtain compound **13** (500.1 mg). F5-9 (2.0 g) was further segmented via Sephadex LH-20 (methanol) to obtain 10 subfractions (F5-9-1~F5-9-10). F5-9-1 (1.0 g) was segmented by CC (silica gel) stepwise gradient elution with trichloromethane-methanol (50:1–10:1) to obtain compound **14** (806.2 mg). F5-10 (5.5 g) was segmented by CC (silica gel) stepwise gradient elution with petroleum ether-acetone (2:1–1:1) to obtain compound **23** (20.1 mg).

Fr.6 (54.0 g) was firstly subjected to reversed-phase RP-18 silica gel eluting with methanol-water (60–100%) to obtain 7 subfractions (F6-1~F6-7). F6-5 (25 g) was segmented by CC (silica gel) stepwise gradient elution with trichloromethane-methanol (100:1–50:1) to obtain compound **13** (10.0 g) and compound **20** (801.5 mg). F6-5-5 (511.6 mg) was segmented by CC (silica gel) stepwise gradient elution with petroleum ether-ethyl acetate (3:1–1:1) to obtain 2 subfractions (F6-5-5-1~F6-5-5-2). F6-5-5-2 (105.3 mg) was further segmented via Sephadex LH-20 (methanol) to obtain compound **24** (89.5 mg).

Fr.7 (43.0 g) was segmented by CC (silica gel) stepwise gradient elution with dichloromethane-methanol (15:1–1:1) to obtain 7 subfractions (F7-1~F7-7). F7-5 (4.0 g) was further segmented via Sephadex LH-20 (methanol) to obtain 7 subfractions (F7-5-1~F7-5-7). F7-5-3 (203.5 mg) was further segmented via Sephadex LH-20 (methanol) to obtain 3 subfractions (F7-5-3-1~F9-5-3-3). F7-5-3-3 (30.6 mg) was segmented by CC (silica gel) stepwise gradient elution with petroleum ether-acetone (2:1–1:1) to obtain compound **6** (5.2 mg). F7-5-7 (40.9 mg) was segmented by CC (silica gel) stepwise gradient elution with trichloromethane-acetone (15:1–1:1) to obtain compound **25** (30.7 mg).

4′,4′-Dimethoxy-sophvein (**17**): white amorphous powder; ${\left[\alpha \right]}_{D}^{24}$ = + 49.88 (*c* 0.25, MeOH); UV *λ*_max_ (MeOH) nm (log *ε*): 214 (4.74), 229 (4.64), 285 (4.58); IR (KBr) ν_max_: 3456, 2950, 1638, 1400, 1098 cm^−1^; ^1^H NMR (Methanol-*d*_4_, 600 MHz) and ^13^C NMR (Methanol-*d*_4_, 150 MHz), see Table 1; HRESIMS at *m/z* 429.1894 [M+Na]^+^ (calcd for C_22_H_30_O_7_Na^+^, 429.1884).

Sophvein-4′-one (**18**): white amorphous powder; ${\left[\alpha \right]}_{D}^{24}$ = +38.69 (c 0.29, MeOH); UV *λ*_max_ (MeOH) nm (log *ε*): 214 (4.48), 231 (4.06), 286 (3.97); IR (KBr) ν_max_: 3446, 2952, 1635, 1406, 1086 cm^−1^; ^1^H NMR (Acetone-*d*_6_, 600 MHz) and ^13^C NMR (Acetone-*d*_6_, 150 MHz), see Table 1; HRESIMS at *m/z* 361.1650 [M+Na]^+^ (calcd for C_20_H_24_O_6_ Na^+^, 361.1646).

### 3.4. Cell Culture Conditions

Murine macrophages (RAW 264.7) were cultured in Dulbecco’s modified Eagle’s medium (DMEM) supplemented with the addition of 10% fetal bovine serum (FBS) and 1% streptomycin (10,000 μg/mL)-penicillin (10,000 U/mL) at 37 °C in a humidified incubator under 5% CO_2_. HepG2 cells were cultured at 37 °C under 5% CO_2_. The optimal fermentation culture medium comprised DMEM, 1% mixed penicillin (10,000 U mL^−1^), 1% HEPES (BioFroxx, Hesse, Einhausen), and 10% fetal bovine serum (FBS), as well as streptomycin (SM) (10,000 μg/mL^−1^) fluid. In all experiments, cells were left to acclimate for 24 h before any treatments.

### 3.5. Cell Viability Examination

To measure cell viability, an MTT assay was performed [41]. Macrophages (4 × 10^4^ per well) were cultured in 96-well plates for 24 h; then, they were treated with several concentrations of compounds **1**–**36** (3.12, 6.25, 12.5, 25, and 50 μM) for 1 h, and then they were stimulated with LPS (1 μg/mL) for another 24 h. After washing twice with a PBS buffer, 20 μL of the MTT solution was added to each well, and incubation continued for 4 h. Optical density (O.D) was measured at 490 nm by a microplate reader (Thermo Fisher Scientific, Waltham, MA, USA). The relative cell viability was calculated in contrast to the normal control group.

### 3.6. NO Production Measurement

Activated macrophage cells could express inducible nitric oxide synthase (iNOS), catalyzing the production of NO from L-arginine. To determine the effect of compounds **1**–**36** on treatments on NO production, the accumulation of nitrites (NO^2−^) in the culture medium was recorded as an indicator of NO production [42,43]. Macrophages (8 × 10^4^ per well) were seeded onto 96-well plates and pretreated by compounds **1**–**36** (3.12, 6.25, 12.5, 25, and 50 μM) 1 h prior to treatment by LPS (1 μg/mL). Afterward, costimulation for 24 h at 37 °C was carried out in an incubator under 5% CO_2_. Then, Griess reagents I and II (100 μL) were mixed with cell culture medium (70 μL). Prior to measuring the optical density, plates were incubated at room temperature for 10 min, and the absorbance at 540 nm was measured using a Thermo Fisher Scientific microplate reader. N(G)-monomethyl-L-arginine, monoacetate salt (L-NMMA), and the medium were used as positive and negative controls, respectively.

### 3.7. Antiproliferative Assay

Briefly, the cells were incubated in 96-well microplates (1 × 10^5^ per well) and allowed to adhere for 24 h before drug administration. Then, the cell lines were treated with test compounds **1**–**36** at five concentrations (3.12, 6.25, 12.5, 25, and 50 μM). At 24 and 48 h of incubation, cells were treated with MTT (200 µL, 5.0 mg/mL) and dissolved in the culture medium, for 1 h under at 37 °C under a 5% CO_2_ humidified atmosphere. The MTT was then removed carefully and resolved with DMSO (150 μL/well). Optical density was recorded using a Thermo Fisher Scientific microplate reader at 490 nm. Cisplatin (CP) and medium were used as positive and negative controls, respectively.

### 3.8. Statistical Analysis

The data were analyzed using Statistical Package for Social Sciences (SPSS Version 21.0) software and are presented as the mean ± S.D. values of three different experiments. The results were analyzed via one-way analysis of variance (ANOVA), and statistical significance was defined as *p* < 0.05.

## 4. Conclusions

In summary, the systematic phytochemical study on the roots of *S. flavescens* led to the isolation of 36 flavonoids (**1**–**36**), including 18 dihydroflavonoids (**1**–**18**), 7 flavonols (**19**–**25**), 4 dihydroflavonols (**26**–**29**), 3 isoflavones (**30–32**), and 4 chalcones (**33**–**36**), identified by spectroscopic methods (^1^H, ^13^C NMR, HSQC, ^1^H-^1^H COSY, HMBC, HRESIMS), and chemical and physical methods. Bioactivity assays indicated that nine compounds exhibited the most significant NO inhibitory activity, better than that of the L-NMMA positive control (IC_50_ = 21.8 ± 0.9 μM). Furthermore, the antiproliferative activities against the HepG2 hepatoma cell lines of all isolates were assessed using the MTT method. The results demonstrated that compound **22** exhibited significant cytotoxic activity, with an IC_50_ value of 0.46 ± 0.1 μM (positive control cisplatin: IC_50_ = 24.5 ± 0.8 μM). This study lays the foundation for studying the potential therapeutic applications of flavonoid derivatives from *S. flavescens* for inflammatory diseases and hepatoma.

## Figures and Tables

**Table 1:** ^1^H and ^13^C NMR data of compounds **17** and **18** ^a^.

Position	17 ^b^		18 ^c^	
	*δ*_H_ (*J* in Hz)	*δ*_C_	*δ*_H_ (*J* in Hz)	*δ*_C_
2	4.04 (d, 13.6)	84.6	4.40 (dd, 13.6, 2.7)	83.7
3a	2.81 (dd, 17.0,13.6)	37.0	3.02 (dd, 17.0, 2.7)	36.9
3b	2.51 (d, 17.0)		2.68 (dd,17.0, 2.7)	
4	–	198.8	–	198.0
5	–	163.0	–	162.8
6	5.84 (s)	96.4	6.01 (s)	96.3
7	–	166.0	–	164.8
8	–	109.0	–	108.1
9	–	161.3	–	160.7
10	–	103.2	–	103.0
1′	–	72.2	–	71.3
2′a	1.75 (m)	29.9	2.21 (m)	36.7
2′b	1.67 (m)		2.21 (m)	
3′a	1.86 (m)	28.4	2.39 (m)	33.0
3′b	1.86 (m)		2.03 (m)	
4′	–	101.1	–	210.3
5′a	1.82 (m)	28.5	2.74 (m)	36.8
5′b	1.65 (m)		2.74 (m)	
6′a	1.72 (d,11.7)	31.3	2.10 (m)	34.0
6′b	1.45 (d,11.7)		2.10 (m)	
1a″	3.14 (d, 6.9)	22.4	3.24 (d, 6.9)	22.1
1b″	3.14 (d, 6.9)		3.24 (d, 6.9)	
2″	5.11 (br t, 6.9)	124.4	5.16 (br t, 6.9)	124.1
3″	–	131.6	–	131.0
4″	1.59 (s)	25.9	1.62 (s)	25.8
5″	1.68 (s)	18.1	1.71 (s)	17.9
4′-OMe	3.15 (s)	47.9	–	–
4′-OMe	3.10 (s)	48.1	–	–

^a^ Assignments were supported with HSQC, HMBC, and ^1^H-^1^H COSY experiments. ^b^ Measured in methanol-*d*_4_ at 600 MHz for ^1^H NMR and at 150 MHz for ^13^C NMR. ^c^ Measured in acetone-*d*_6_ at 600 MHz for ^1^H NMR and at 150 MHz for ^13^C NMR.

**Table 2:** Inhibitory effects of compounds **1**–**36** on LPS-induced NO production in macrophages (IC_50_ values less than 20 μM are listed).

Compds	IC_50_^b^ (μM)	CC_50_^c^ (μM)	Compds	IC_50_^b^ (μM)	CC_50_^c^ (μM)
**2**	8.4 ± 0.7	>50	**22**	5.7 ± 0.4	13.8 ± 0.6
**12**	11.2 ± 1.1	25.7 ± 1.1	**29**	7.2 ± 0.6	20.6 ± 1.1
**13**	14.4 ± 0.4	33.8 ± 1.0	**35**	4.6 ± 1.1	21.9 ± 0.4
**15**	9.3 ± 0.7	18.8 ± 0.3	**36**	6.7 ± 0.6	16.2 ± 0.9
**19**	12.4 ± 0.9	>50	L-NMMA ^a^	21.8 ± 0.9	>50

^a^ L-NMMA was used as positive control. ^b^ IC_50_: 50% inhibitory concentration. (Mean ± SD of 3 tests.) ^c^ CC_50_: 50% cytotoxic concentration. (Mean ± SD of 3 tests.)

**Table 3:** Antiproliferative effects of compounds **1**–**36** on HepG2 cell lines (IC_50_ values less than 50 μM are listed).

Compounds	IC_50_^b^ (μM)	Compds	IC_50_^b^ (μM)
**1**	34.9 ± 0.3	**21**	26.4 ± 0.3
**5**	37.6 ± 0.7	**22**	0.46 ± 0.1
**16**	30.8 ± 1.1	**34**	48.6 ± 0.8
**19**	41.4 ± 0.6	**35**	46.2 ± 0.7
Cisplatin ^a^	24.5 ± 0.8	-	-

^a^ Cisplatin was used as positive control. ^b^ IC_50_: 50% inhibitory concentration. (Mean ± SD of 3 tests.).

## Data Availability

The data presented in this study are available in the Supplementary Materials.

## Supplementary Materials

The following supporting information can be downloaded at: https://www.mdpi.com/article/10.3390/molecules28052048/s1, Figures S1–S14 HRESIMS, ^1^H and ^13^C NMR, HMQC, HMBC, ^1^H-^1^H COSY, and ROESY of compounds **17** and **18**.

## Sample Availability

Samples of the compounds are not available from the authors.

## Abbreviations

CC, chromatograph column; COSY, correlation spectroscopy; DEPT, distortionless enhancement by polarization transfer; HPLC, high-performance liquid chromatography; HR-ESI-MS, high-resolution electrospray ionization mass spectroscopy; HMBC, heteronuclear multiple bond correlation; HSQC, heteronuclear single quantum coherence; L-NMMA, N(G)-monomethyl-L-arginine; LPS, lipopolysaccharide; MPLC, medium-pressure liquid chromatography; NMR, nuclear magnetic resonance; MS, mass spectrometry; NO, nitric oxide; NOE, nuclear overhauser effect; ROESY, rotating-frame overhauser effect spectroscopy; TCM, traditional Chinese medicine; TLC, thin-layer chromatography; TMS, tetramethylsilane.
