# Supplementary material for: Flavonoids from the Roots of Sophora flavescens and Their Potential Anti-Inflammatory and Antiproliferative Activities
Source: Molecules. 2023 Feb 22;28(5):2048. doi: 10.3390/molecules28052048 (PMC10004487; doi:10.3390/molecules28052048)
Supplement: Supplementary file 1 [file molecules-28-02048-s001.zip › molecules-2196555-supplementary.pdf]

Flavonoids from the Roots of *Sophora flavescens* and Their Potential Anti-inflammatory and Antiproliferative Activities

Yan-Fei Yang[†],Ting-Ting Liu[†], Guo-Xian Li[†], Xuan-Qin Chen, Rong-Tao Li[*], Zhi-Jun Zhang[*],

Faculty of Life Science and Technology, Kunming University of Science and Technology, Kunming, 650500, China

[*]Corresponding author: lirt@kust.edu.cn (R.-T. L), zzj0121@126.com (Z.-J. Z);

[†]These authors contributed equally to this work.

Table of contents	Pages
**Figure S1.** HRESIMS of **17**	2
**Figure S2.** ^{1}H NMR spectra of **17**	3
**Figure S3.** ^{13}C and DEPT NMR spectra of **17**	4
**Figure S4.** HSQC spectrum of **17**	5
**Figure S5.** HMBC spectrum of **17**	6
**Figure S6.** COSY spectrum of **17**	7
**Figure S7.** ROESY spectrum of **17**	8
**Figure S8.** HRESIMS of **18**	9
**Figure S9.** ^{1}H NMR spectra of **18**	10
**Figure S10.** ^{13}C and DEPT NMR spectra of **18**	11
**Figure S11.** HSQC spectrum of **18**	12
**Figure S12.** HMBC spectrum of **18**	13
**Figure S13.** COSY spectrum of **18**	14
**Figure S14.** ROESY spectrum of **18**	15

Qualitative Analysis Report

**Data File**	fugx26.d	**Sample Name**	fugx26
**Sample Type**	Sample	**Position**	P1-A1
**Instrument Name**	Instrument 1	**User Name**	6530-HP\6530
**Acq Method**	20201225-HRMS2min.m	**Acquired Time**	12/30/2020 12:05:05 PM
**IRM Calibration Status**	Success	**DA Method**	Default.m
**Comment**			
**Sample Group**		**Info.**	
**Stream Name**	LC 1	**Acquisition SW Version**	6200 series TOF/6500 series Q-TOF B.06.01 (B6172 SP1)

User Spectra

Fragmentor Voltage	Collision Energy	Ionization Mode
175	0	ESI

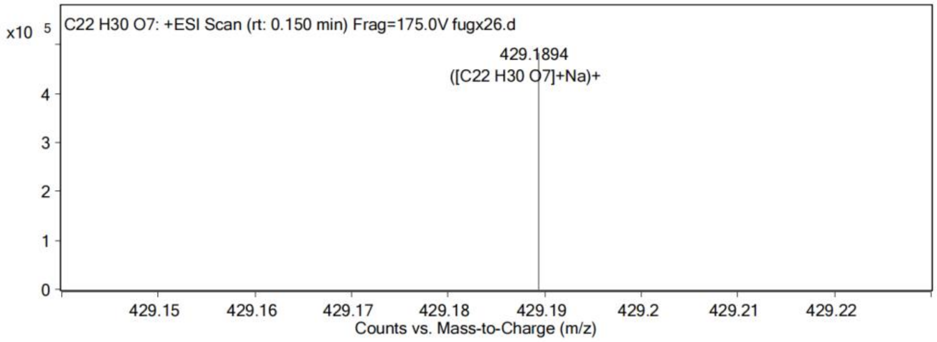

Formula Calculator Element Limits

Element	Min	Max
C	3	60
H	0	120
O	0	30

Formula Calculator Results

Formula	Best	Mass	Tgt Mass	Diff (ppm)	Ion Species	Score
C22 H30 O7	True	406.2002	406.1992	-2.46	C22 H30 Na O7	45.24

--- End Of Report ---

Agilent Technologies Page 1 of 1 Printed at 5:49 PM on 30-Dec-2020

Figure S1. HRESIMS of **17**

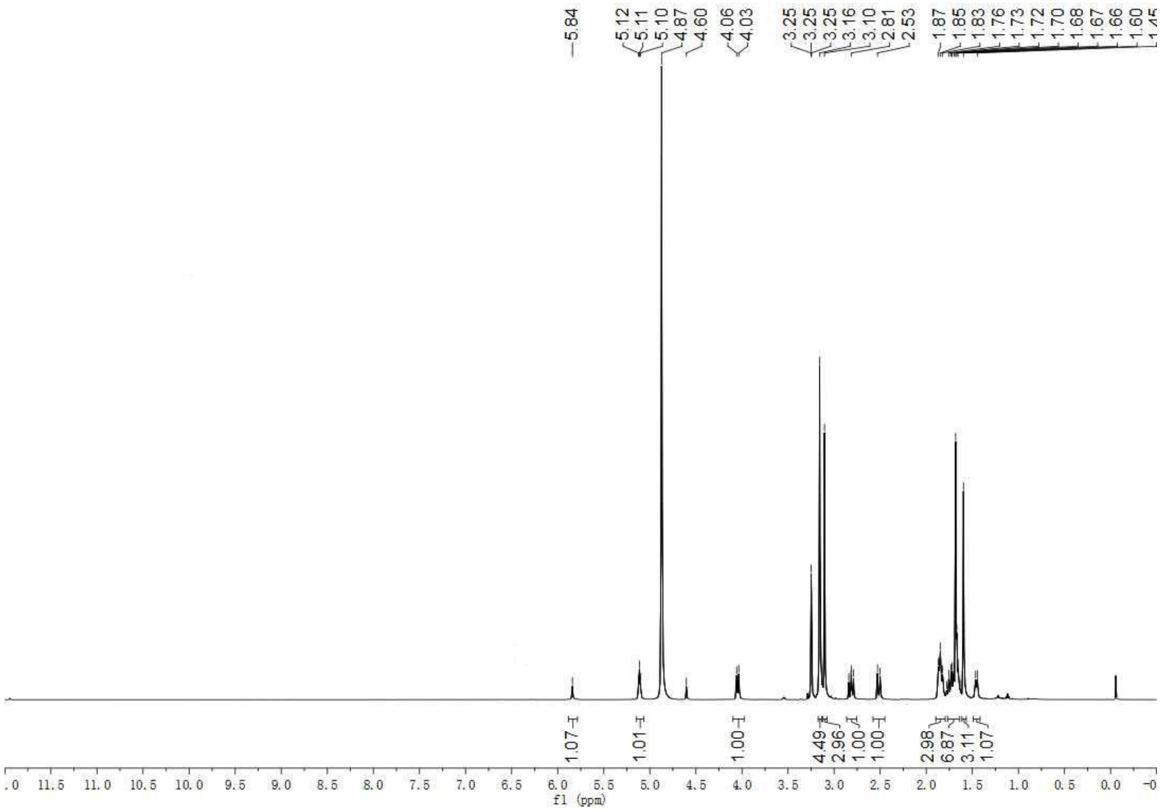

Figure S2. ^{1}H NMR spectra of **17**

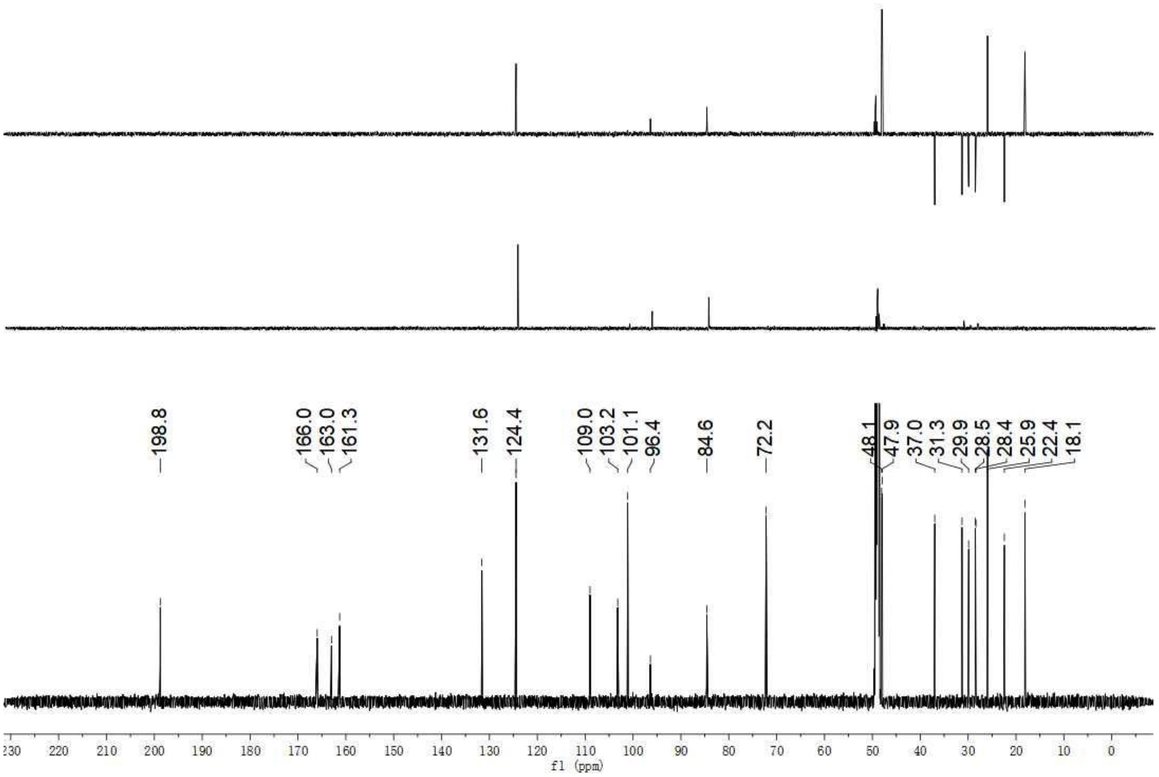

Figure S3. ^{13}C and DEPT NMR spectra of **17**

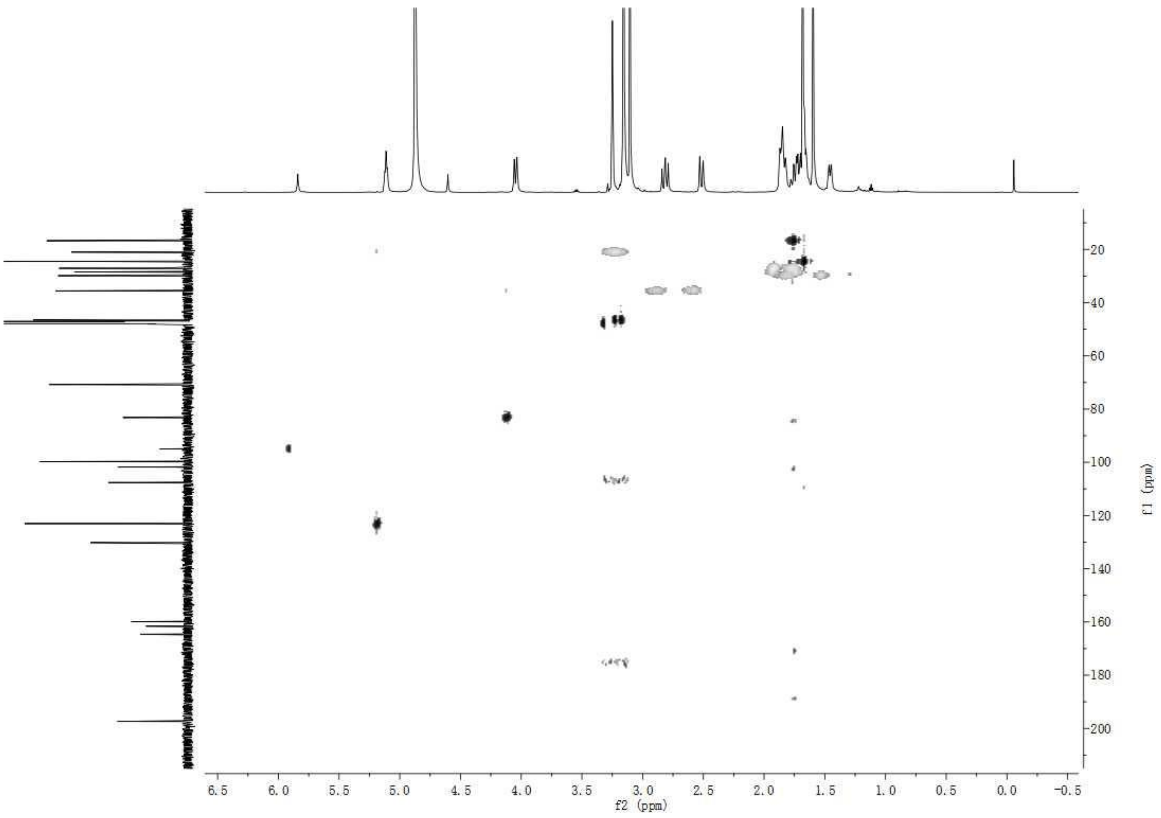

Figure S4. HSQC spectrum of **17**

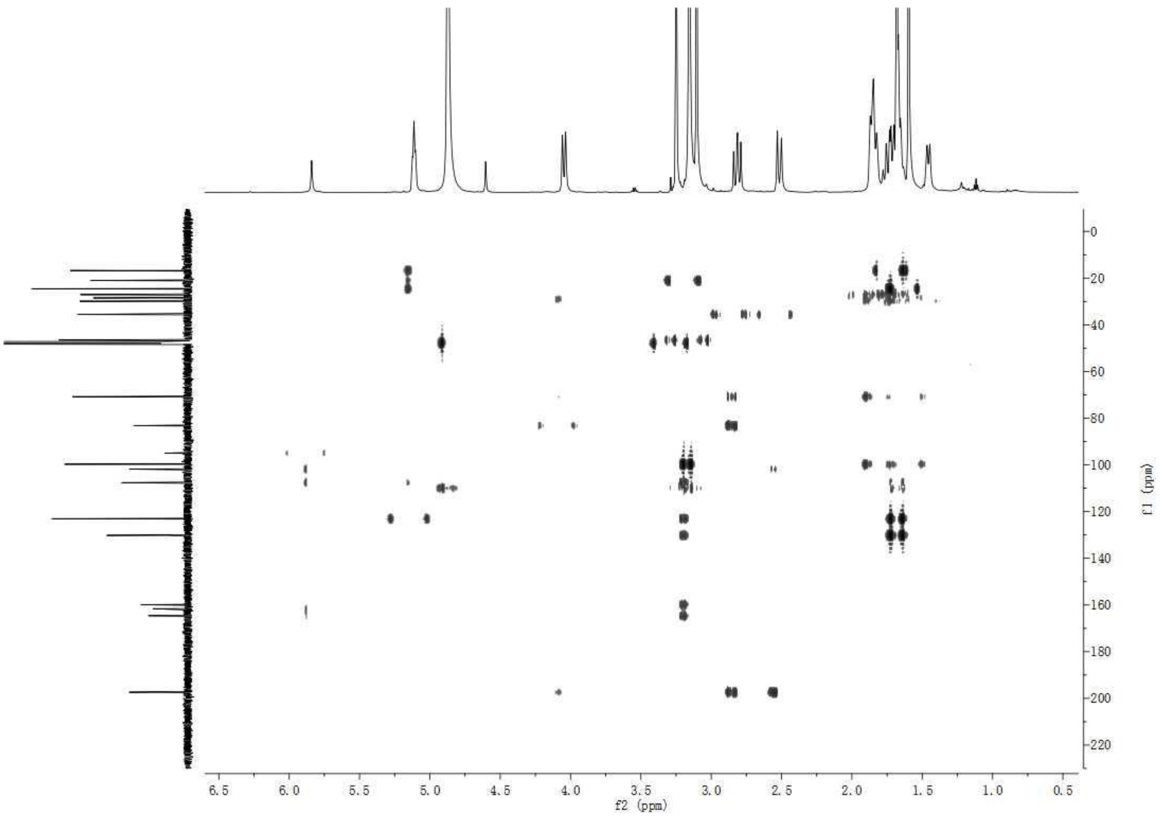

Figure S5. HMBC spectrum of **17**

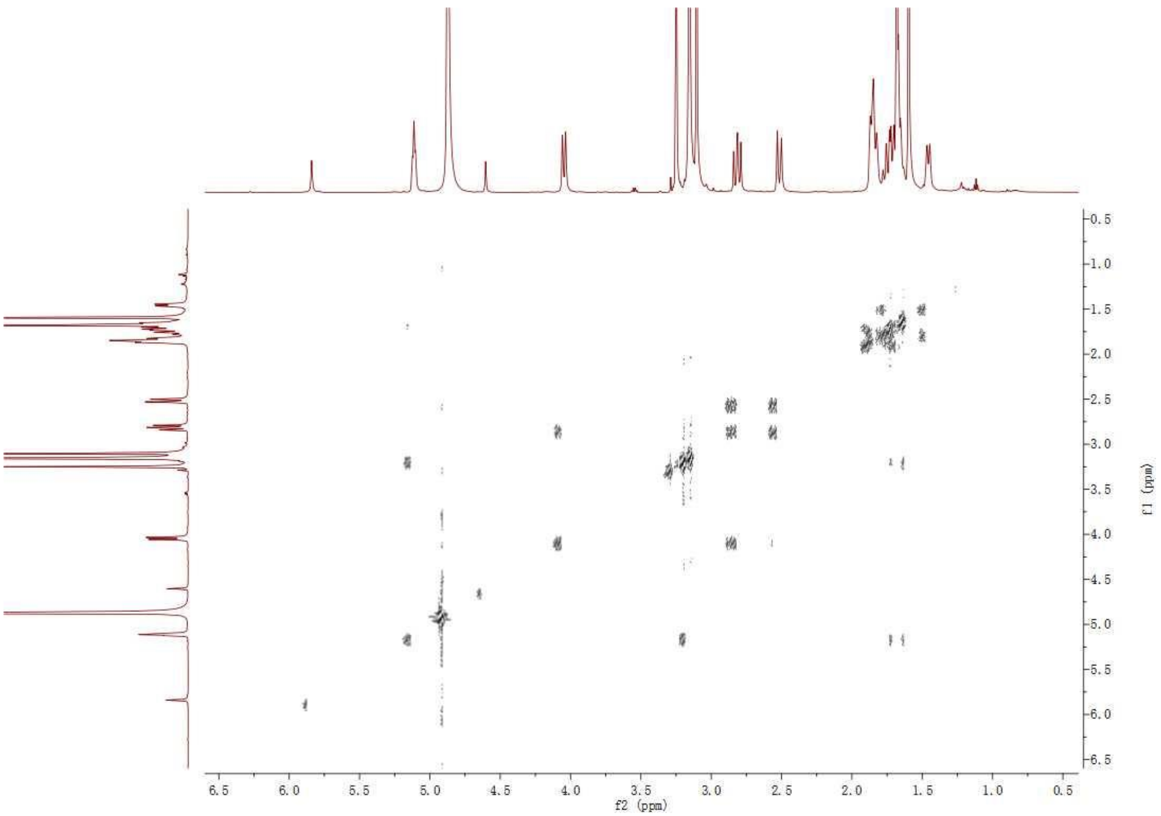

Figure S6. COSY spectrum of **17**

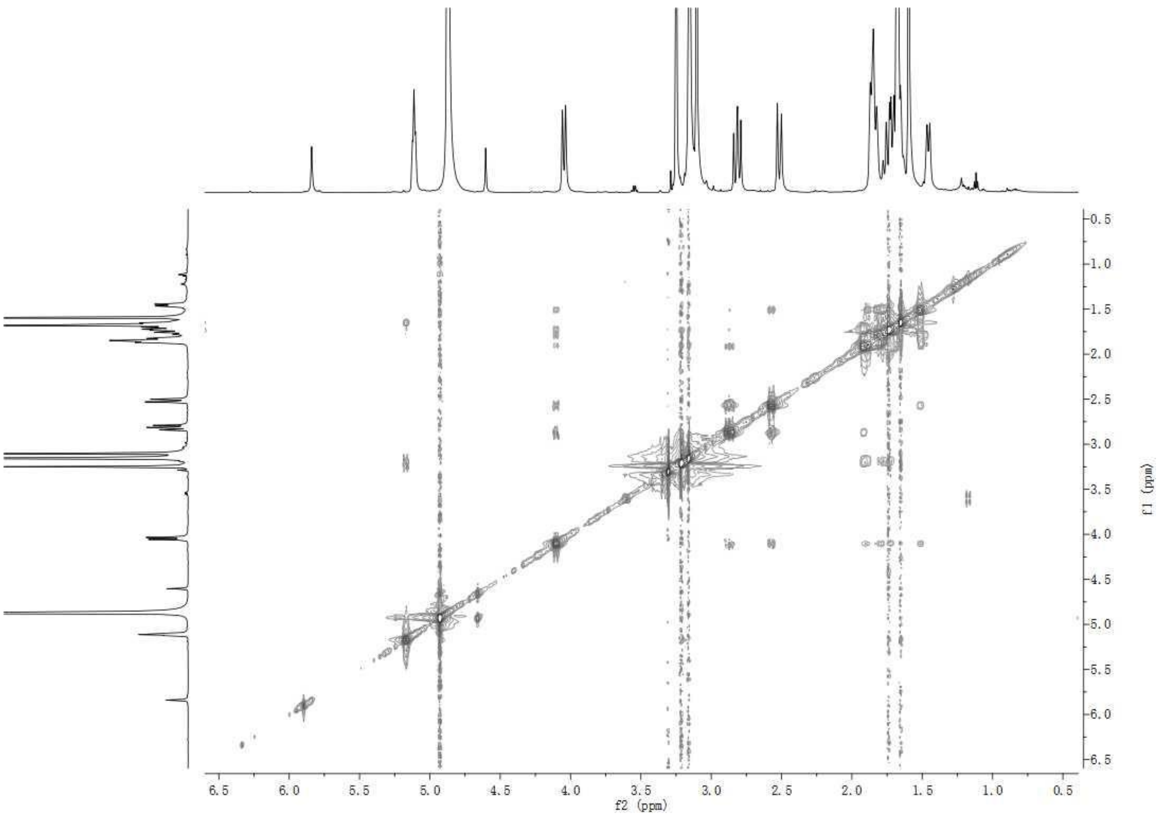

Figure S7. ROESY spectrum of **17**

Qualitative Analysis Report

**Data File**	fugx28.d	**Sample Name**	fugx28
**Sample Type**	Sample	**Position**	P1-A1
**Instrument Name**	Instrument 1	**User Name**	6530-HP\6530
**Acq Method**	20201225-HRMS2min.m	**Acquired Time**	12/30/2020 10:56:47 AM
**IRM Calibration Status**	Success	**DA Method**	Default.m
**Comment**			
**Sample Group**		**Info.**	
**Stream Name**	LC 1	**Acquisition SW Version**	6200 series TOF/6500 series Q-TOF B.06.01 (B6172 SP1)

User Spectra

Fragmentor Voltage	Collision Energy	Ionization Mode
175	0	ESI

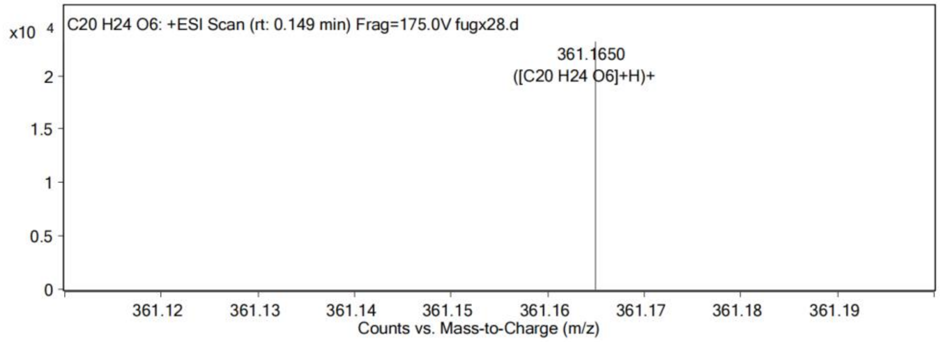

Formula Calculator Element Limits

Element	Min	Max
C	3	60
H	0	120
O	0	30

Formula Calculator Results

Formula	Best	Mass	Tgt Mass	Diff (ppm)	Ion Species	Score
C20 H24 O6	True	360.1578	360.1573	-1.42	C20 H25 O6	83.12

--- End Of Report ---

Figure S8. HRESIMS of **18**

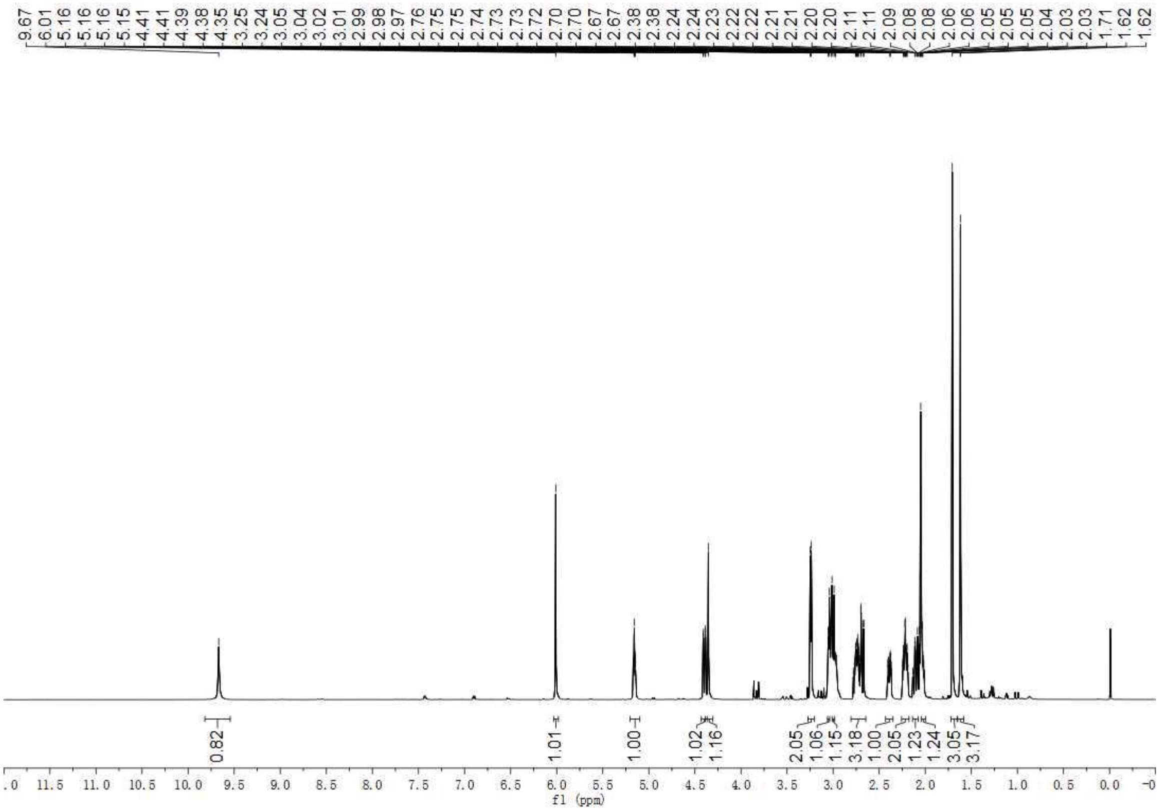

Figure S9. ^{1}H NMR spectra of **18**

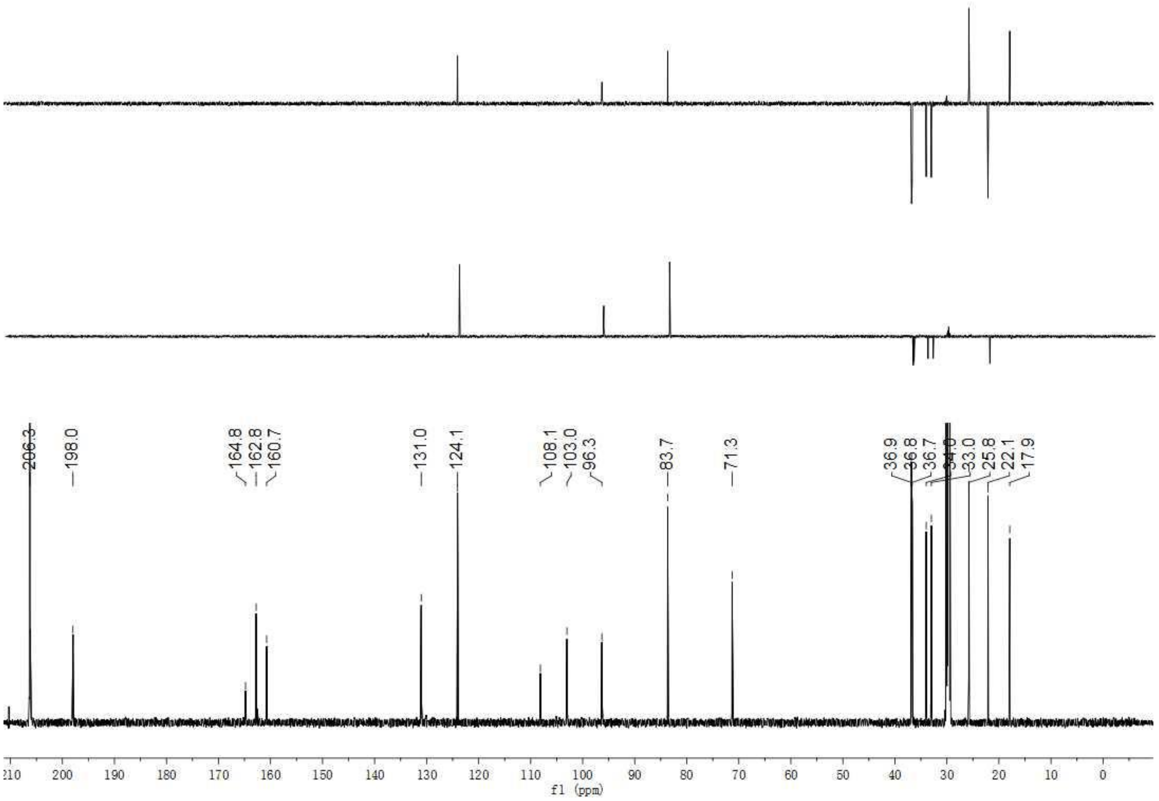

Figure S10. ^{13}C and DEPT NMR spectra of **18**

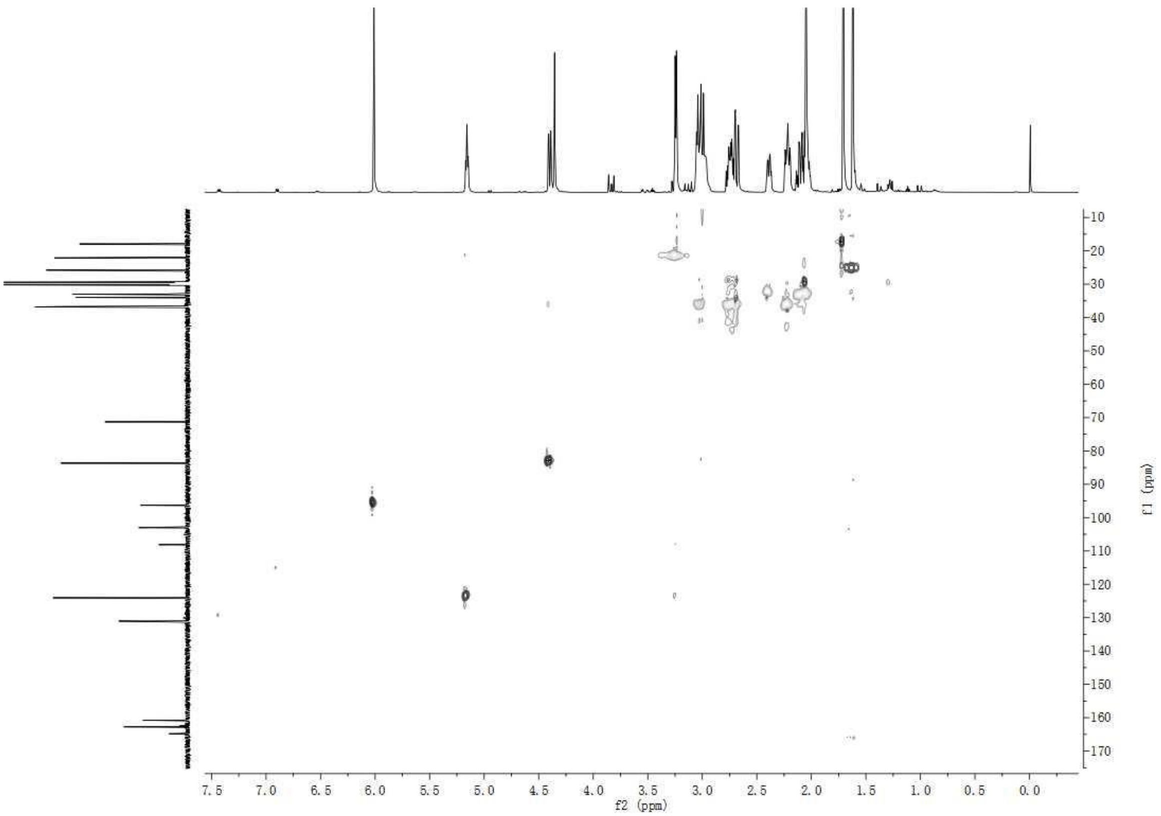

Figure S11. HSQC spectrum of **18**

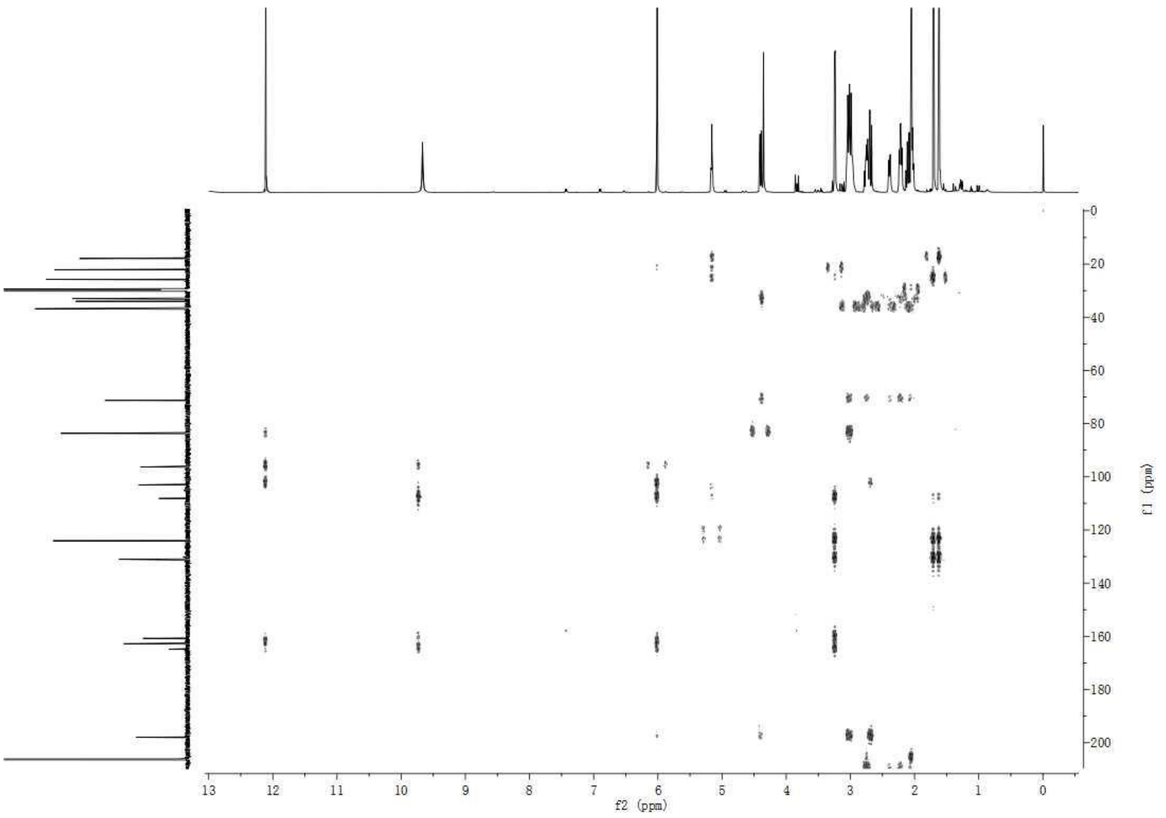

Figure S12. HMBC spectrum of **18**

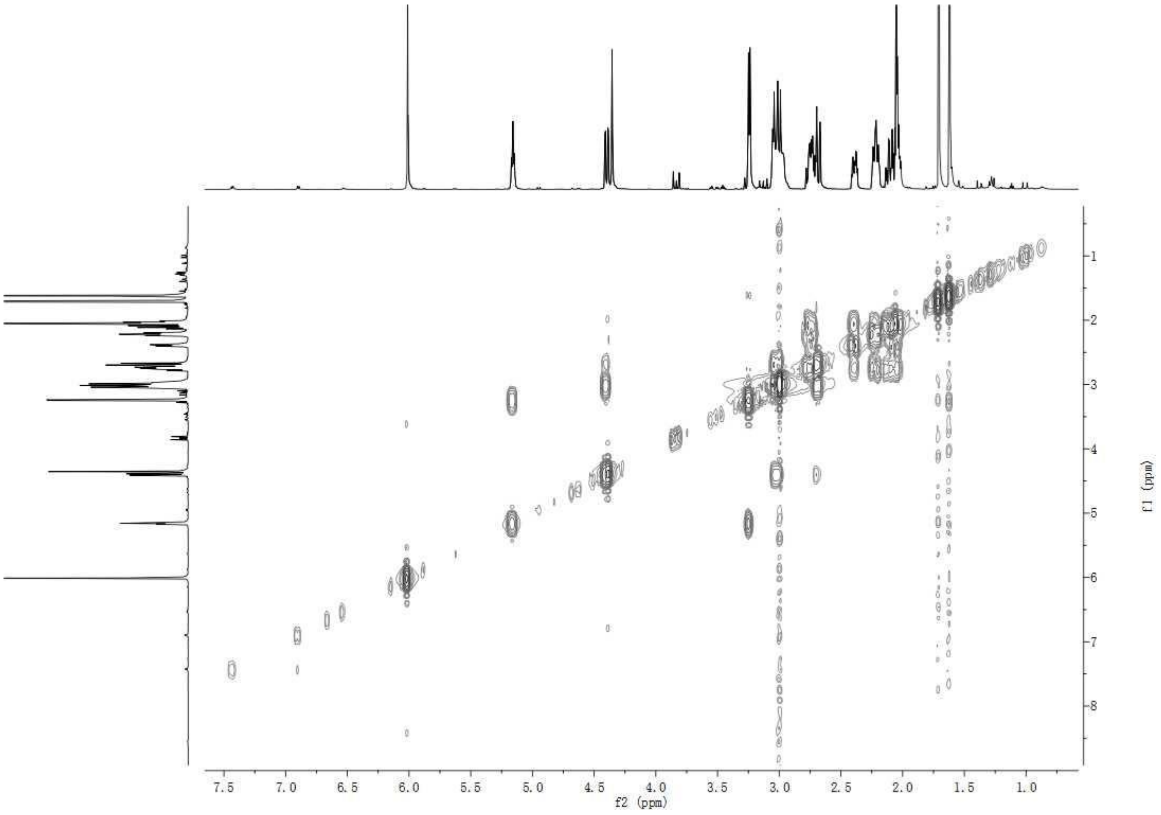

Figure S13. COSY spectrum of **18**

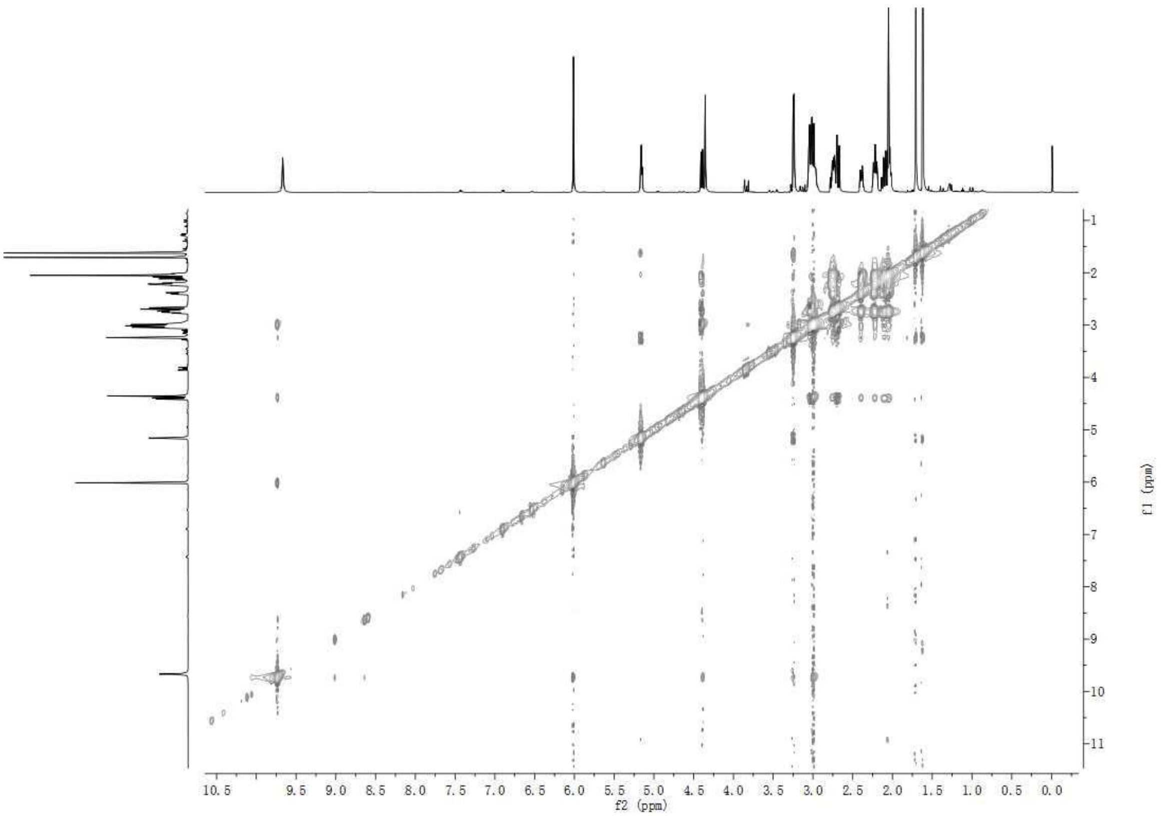

Figure S14. ROESY spectrum of **18**
